# The Dental Obturator

**Published:** 1855-10

**Authors:** A. S. Talbert

**Affiliations:** Lexington, Ky.


### THE DENTAL OBTURATOR.

BY A. S. T. ALBERT, D. D. S. Lexington, Ky.

This is the title of a new Quarterly Journal, to be "devoted to the science and art of dentistry," and edited by John S. Clark, D. D. S., New Orleans.

Commendable as are the objects of this new light to our profession, incubated by the genial rays of the sunny South and fostered by a host of enterprising publishers. Its ostensible object looks ( to a man up the tree ) a little *personal.*

Its modest introductory commences with an originality—(in syntax,) that is truly characteristic of a great *beginning* to write a *book.*

After passing over some fourteen pages, we find the following, which ought to be kept in the archives of the profession, as a specimen both of its *literature* in the "ninteenth century," as well as the *frequent* performance of some of its more difficult operations, to wit: "We do not wish to be understood as wishing to decide as to the manner of operating, but will give a few facts and descriptions of the means by which we have been the most successful; and premise by saying, that on examination of our book of record of operations, we have for several seasons past averaged one per day, and that in all cases when the nerve is attacked by disease the operation of entire extirpation is performed. This is our general rule of practice. The exceptions are in some cases of entirely healthy pulps, on very slight exposure the actual cautery is applied and the pulp saved alive."

Query.—What is the extent of a man's business whose book of record averages per day "one entire extirpation of the nerve?" But this "*actual cautery*"—who is he? I have inquired of Mrs. Partington, who inclined to the opinion that he is one of Napoleon's officers "in the *Crimea*," but when I told her it was something about *burning healthy* pulps in teeth, to "*save them* alive," she only "wished that *pups had no teeth!*"

But aside from all this, it becomes my duty to notice the editorial remarks in relation to a paper read by the writer of this, before the Mississippi Valley Association of Dental Surgeons," and published along with the other proceedings of that body, in the April number of the Dental Register of the West.

I am glad of an opportunity to speak of that article, through the medium in which it was first published, that I may correct the print of one word, which I suppose required to be "read and reread, and read again" by others, as well as the editor of the "Obturator." It was written "protean"—(be it good or bad) instead of "probang" as the Register has it, or "quandam" as the New-York Recorder has it.

It may be further remarked that, that paper was not *writ,-ten* for *publication*, but to be read before the association, and blunt points and careless expressions were thrown in on purpose to excite discussion, which I suppose to be the object which that body has in view in making its annual appointments.

The editor of the Obturator seems quite at a loss to understand the simple instrument described in that paper as a "Hard drill,"—and after attempting its description he calls it a "screw driver." Now, however my description may have failed to give a correct idea of its shape, it is not like a "screw driver," and I challenge him or any other "worker in hard metals" to "produce a better cutting point in an instrument that shall fill its place." "But what shall we say of its temper." "Hard as fire and water can make it"—this was merely an expression of mine to indicate extreme hardness, without reference to the manner of *obtaining* the required results. It was presumed that the association were aware of the results that would be produced by instituting the experiments proposed by the learned editor in "drawing the temper" in hardened instruments.

The object is not to procure an elastic cutting edge but a *hard edge*, and fire and water will do this about as efficiently as any other means. Their shape secures them against breaking especially the larger ones, the smaller being drawn to the various shades of "straw color," as would naturally be suggested to the operator by their tendency to break, notwithstanding their comparative inefficiency when thus temperd a fact deemed unworthy of note in the original paper.

The inutility of a great variety of shapes in excavators is granted in the editoral, in review, hence I shall be no more molested in "hatchet and hoe monopoly" but may take them up and "lay *em* down at pleasure."

But that "peculiar protest," about which so much is said, and which involves such sublime interests to our profession. I confess it is a subject worthy a separate paper, and I will not degrade it by introducing it into a conflict so puerile as this little matter between myself and the editor in question; but begging the pardon of your readers, I will indorse his sentiment of "distaste for controversy," nor should I have noticed his remarks and "*mistakes*," had they not been "promulgated under cloak of (editoral) authority."

